# Impacts of forestation and deforestation on local temperature across the globe

**DOI:** 10.1371/journal.pone.0213368

**Published:** 2019-03-20

**Authors:** Jayme A. Prevedello, Gisele R. Winck, Marcelo M. Weber, Elizabeth Nichols, Barry Sinervo

**Affiliations:** 1 Department of Ecology, Institute of Biology Roberto de A. Gomes, Rio de Janeiro State University, Rio de Janeiro, RJ, Brazil; 2 Department of Ecology, Institute of Biology, Federal University of Rio de Janeiro, Rio de Janeiro, RJ, Brazil; 3 Biology Department, Swarthmore College, Swarthmore, PA, United States of America; 4 Department of Ecology, Instituto de Biologia, Universidade de São Paulo, São Paulo, SP, Brazil; 5 Department of Ecology and Evolutionary Biology and the Institute for the Ecological and Evolutionary Study of Climate Impacts, University of California, Santa Cruz, CA, United States of America; Texas A&M University, UNITED STATES

## Abstract

Changing forest cover is a key driver of local climate change worldwide, as it affects both albedo and evapotranspiration (ET). Deforestation and forestation are predicted to have opposing influences on surface albedo and ET rates, and thus impact local surface temperatures differently. Relationships between forest change, albedo, ET, and local temperatures may further vary regionally, as the strengths of warming by albedo effects and cooling by ET effects vary with latitude. Despite these important relationships, the magnitude of forest cover effects on local surface temperature across the globe remains unclear. Using recently-released global forest change data, we first show that forestation and deforestation have pervasive and opposite effects on LST, ET and albedo worldwide. Deforestation from 2000 to 2010 caused consistent warming of 0.38 ± 0.02 (mean ± SE) and 0.16 ± 0.01°C in tropical and temperate regions respectively, while forestation caused cooling in those regions of -0.18 ± 0.02 and -0.19 ± 0.02°C. Tropical forests were particularly sensitive to the climate effects of forest change, with forest cover losses of ~50% associated with increased LST of 1.08 ± 0.25°C, whereas similar forest cover gains decreased LST by -1.11 ± 0.26°C. Secondly, based on a new structural equation model, we show that these changes on LST were largely mediated by changes in albedo and ET. Finally, based on this model, we show that predicted forest changes in Brazil associated with a business-as-usual land use scenario through 2050 may increase LST up to 1.45°C. Our results contribute to a better understanding of the mechanistic inter-relationships between forest change and changes in albedo, ET and LST, and provide additional evidence that forestation has the potential to reverse deforestation impacts on local climate, especially in tropical and temperate regions.

## Introduction

Forests originally covered 40% of Earth's terrestrial surface [1], but extensive deforestation over the past 300 years has reduced this area substantially (e.g. [2]). On the other hand, forestation (i.e., forest cover increase) is also common globally, and reflects both the passive regeneration of vegetation (e.g., in abandoned agriculture land) and the active restoration and planting of new forests [3]. For example, planted forests increased in extent from 16.7 million km^2^ in 1990 to 27.8 million km^2^ in 2015 [4]. From 2000 to 2012, forest change—either deforestation or forestation—occurred across 3.1 million km^2^ globally [5]. Forest cover change has widespread social, economic, and ecological consequences, as forests affect the provisioning of ecosystem services, the integrity of biological communities, as well as climate and air quality [6–9]. Until recently however, the limited availability of high-resolution forest data has hampered quantification of forest change impacts at a global scale [5,10–13].

One under-examined environmental consequence of this global forest change is the modification of local-scale climate (e.g., 5 x 5 km areas). Forests may exert a strong influence on local land surface temperature (LST), as they generally have lower surface albedo and higher evapotranspiration (ET) compared to open vegetation physiognomies [10–15]. Reduced albedo can induce warming through higher absorption of shortwave radiation, however this effect may be offset by the loss of latent heat via higher ET [12,15,16]. The strength of both albedo warming and ET cooling further varies with latitude [15], suggesting that the magnitude and even the direction of forest change effects on local LST may vary among tropical, temperate, and boreal regions [11,15,17]. Recent studies have quantified how some biophysical processes, such as shortwave radiation and latent heat flux (directly coupled to albedo and ET, respectively), vary latitudinally, and influence surface temperature in response to forest change [10–13,18]. Yet, it remains unclear how the hierarchical inter-relationships between forest change, albedo, ET, and LST vary across the globe.

Additional uncertainty arises from the degree to which local forestation may counteract the effects of local deforestation on LST, albedo and ET. Only a few recent studies have compared forestation and deforestation impacts on local climate [10,11]. About 20 million km^2^ of land are available for forest restoration worldwide, and a global effort intends to restore 3.5 million km^2^ by 2030 [19]. Concurrently, forest losses exceeding 15% of global forest cover are also predicted by 2030 [20]. Importantly, such changes are likely to affect local climate across the Earth’s surface in ways that may exacerbate projected regional and global climate changes [21].

Here we use a recent high-resolution dataset on global forest change from 2000 to 2010 [5] to quantify impacts of past forest change on LST, albedo and ET across the globe, as well as the hierarchical inter-relationships between these variables. We used multilevel structural equation modeling [22] to (i) quantify the presumed causal relationships among forest change and changes in albedo, ET, and LST, and (ii) assess how these relationships vary across regions (tropical, temperate, and boreal). We also use our structural models to illustrate how future (2010–2050) forest change may affect local LST in Brazil. Brazil has a continental size (8.5 million km^2^) and is undergoing severe changes in land use patterns [23]. Furthermore, Brazil harbors two hotspots of biodiversity (Atlantic Forest and Cerrado) and the Amazon, the largest tropical forest in the world.

## Materials and methods

To quantify impacts of past forest change on local climate, we adopted a six-step methodological approach, as detailed in the following sections. These steps included (i) compilation of global forest and climate datasets for two time periods (2000/2010 and 2001/2011); (ii) preliminary treatment of all datasets, including quality control, standardization of spatial resolution and calculation of annual averages; (iii) calculation of forest change from 2000 to 2010 across the globe; (iv) calculation of the change in each climatic variable (LST, albedo, and ET) across the same 2000 to 2010 period; (v) application of a “window searching” strategy to compare forest and climate change between pairs of close cells (< 25 km apart); and (vi) application of statistical analyses to quantify causal relationships among ΔF, Δalbedo, ΔET, and ΔLST. To show how our approach can be further applied, we then model future changes in local climate in Brazil, by following four main steps: (i) application of the window searching algorithm to past (2000–2010) forest change data for Brazil only; (ii) fitting of a general structural equation model (i.e., path model) to these data and extraction of model coefficients; (iii) compilation of future (2010–2050) forest change data for Brazil; and (iv) calculation of predicted changes in LST across Brazil. We performed all data processing and analysis in R 3.3.2 [24].

### Forest and climate data

We obtained forest cover data for the years 2000 and 2010 from the 30-meter Global Forest Cover datasets provided by Hansen *et al*. [5] (see also https://landcover.usgs.gov/glc/). The datasets for both years are derived from Landsat 7 ETM+ data and cover most global land (128.8 Mkm^2^). The datasets comprise the tree cover estimates for each 30-m pixel, varying from 0 to 100%, and include both natural and planted trees (i.e., vegetation taller than 5m). We assume that tree cover represents forest cover as in Hansen *et al*. [5]. For our analysis, we upscaled the original maps to a resolution of 0.05° (~ 5 km), by averaging values of the 30-meter pixels, to match MODIS LST data (see below). The 5 x 5 km cells represented the “local” scale of analysis, both for forest cover and for the climatic variables (LST, ET, and albedo). We quantified forestation and deforestation as an increase or decrease in forest cover from 2000 to 2010, respectively, at each 5 x 5 km cell across the globe.

We obtained three climatic variables—land surface temperature (LST), evapotranspiration (ET) and albedo—from collection-5 MODIS products [25], with overpass time at 10:30 and 22:30 hours, which have been extensively validated and used in many previous applications (e.g. [16,26]). We gathered all climatic variables (LST, ET and albedo) for the years 2001 and 2011, rather than 2000 and 2010 as we did for the forest change. This choice reflected our expectation that forest change (cause) would precede climate change (consequence). We found similar results when using 2000 and 2010 climate data (S1 Fig), although with larger variability in the results, reflecting higher heterogeneity of the forest cover maps, as they were produced combining satellite imagery collected at different months of 2000 and 2010 [5].

We obtained monthly daytime and nighttime LST (in°C) at 0.05° resolution from the MOD11C3 product. For quality control, we only used LST data with estimated emissivity error ≤ 0.02 and LST error ≤ 2 K (the original temperature unit for this dataset). We averaged data across both day and nighttime, as well as across months to obtain a single average, hereafter referred to as annual LST. We present in the main text the results for annual LST only, as our primary aim was to assess the overall effects of forestation and deforestation on local temperature. However, we also present the separate results for daytime and nighttime LST in the supplementary material.

We obtained ET data (in mm/month) from the MOD16A3 product, at 1-km (ca. 0.0083°) resolution. This dataset includes estimates of ET from daily meteorological reanalysis data along with MODIS remotely sensed data, using Mu *et al*.’s algorithm [27], based on the logic of the Penman-Monteith [28]. We upscaled the original values to 0.05° resolution (equivalent to 6 x 6 original pixels) through the bilinear interpolation resampling method, to match the LST and the forest cover data. For quality control of ET data, we only included in the analysis 0.05° pixels for which all the 36 original pixels (0.0083°) contained data. We averaged monthly ET data to obtain a single annual ET estimate for each cell. We obtained white sky albedo data (in %) from the MCD43C3 product, at 8-day intervals and 0.05° resolution. We condensed the 8-d intervals into monthly averages and subsequently into annual averages. We only used albedo cells flagged with 0, 1 or 2 (best, good, and mixed quality, respectively). Many pixels had lower quality for albedo (flagged with 3 or higher), mostly due to cloud cover and were excluded. For comparison, we also considered an alternative MODIS 8-day albedo dataset (“GLASS”; [29, 30]), which returned similar results (S2 Fig). Variable quality of the original products resulted in different numbers of cells for the analysis of LST, ET and albedo (see Data Analysis).

For each 5 x 5 km cell around the globe, we calculated forest change as the percentage forest cover in 2010 minus the percentage forest cover in 2000. We did not consider interannual dynamics of forest cover from 2000 to 2010, only the final outcome of those dynamics, i.e., percentage of forest cover in 2010 minus 2000. Resulting forest change (ΔF) values varied from -100% to +100%, corresponding to maximum deforestation to maximum forestation, respectively, during the one-decade time period. Similarly, we calculated the decadal change in LST, albedo and ET for each cell as the annual average in 2011 minus the annual average in 2001 (ΔLST, Δalbedo and ΔET, respectively). We focused only on annual averages for each climatic value, disregarding seasonal variation, as there are no global forest cover maps available for different seasons within each year. We acknowledge, however, that forest change impacts may vary seasonally, especially in the boreal region, as we mention in the Discussion (see also [15]).

### Window searching strategy

**Fig 1:**
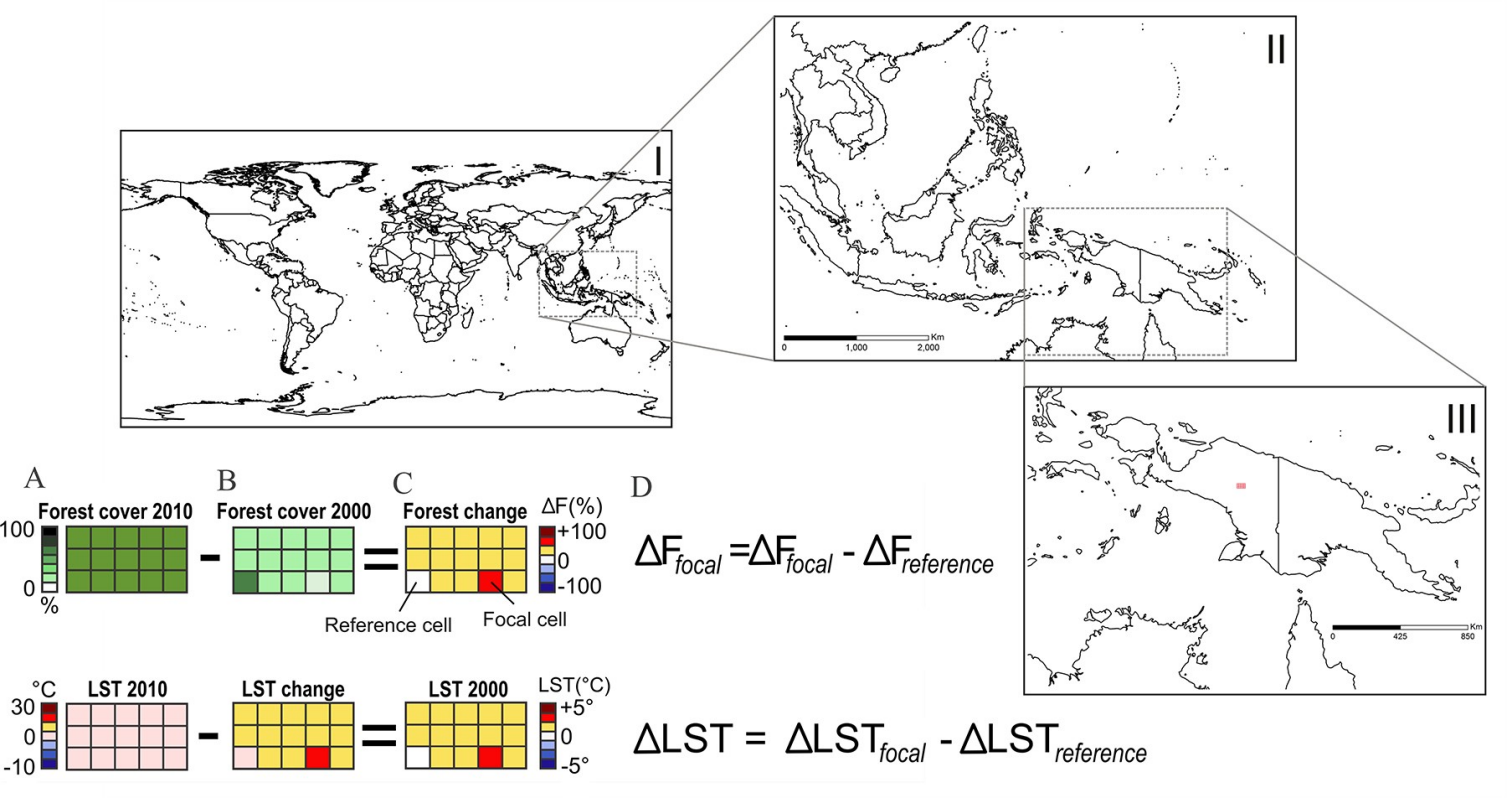
Methodological approach applied to quantify the relationship between forest change and local climate change. This approach was applied worldwide (I) to compare close cells sharing a similar regional climate (II and III). Each window had 15 cells of 0.05° resolution (5 longitude x 3 latitude cells). To facilitate visualization, a single window is depicted (red grid in III). In each window, data on local forest cover and local land surface temperature (LST) were compared by subtracting values in different years (2010–2000 for forest cover, 2011–2001 for climate; A and B), generating a georeferenced matrix of forest change and another of LST change (C). From the resulting matrices, two cells were chosen: a “focal” cell, with absolute forest change > 15%, and a “reference” cell, with forest change < 5%. Finally, we calculated the standardized forest change (ΔF) and the standardized LST change (ΔLST) (D), by subtracting the focal cell values by the reference cell values.

To quantify the relationship between forest change and local climate change around the globe, we applied a “window searching” strategy (Fig 1). This strategy allows comparing pairs of close cells (< 25 km apart) with different amounts of forest change but exposed to a similar regional or “background” climate, and has been used in previous studies [e.g., 15]. Windows had 5 x 3 cells (longitude x latitude), with adjacent windows partially overlapping in 3 cells in longitude and 1 cell in latitude. We found similar results with a larger window (9 x 5 pixels; S3 Fig). We then used a simple algorithm to record forest and climate changes in a single pair of selected cells per window. Each pair was formed by one “focal” cell, i.e., a cell with absolute forest cover change greater than 15%, and one “reference” cell, i.e., a cell with absolute forest cover change lower than 5% (Fig 1). We found similar results when we used > 10% and < 2% as the threshold values for focal and reference cells, respectively (S4 Fig). If more than one focal cell was available within a window, we selected only the cell with greater absolute forest change as the focal cell. This approach did not lead to overestimating forest change impacts, because in most windows all potential focal cells had a relatively low absolute forest change (<25%) Similarly, if more than one reference cell was available within a window, we selected only the cell with lower absolute forest change as the reference cell. This approach allowed us to obtain pairs of cells with varied degrees of difference in forest change, and also ensured a single pair of cells was used within a window, reducing spatial autocorrelation. Finally, if the same pair of selected cells occurred in two adjacent windows, we only used the first occurrence in the subsequent analyses. There was no need to control for differences in elevation between cells of a same pair, as we did not use the raw value (e.g., LST) of each cell in the analysis, but rather the decadal change in cell value (e.g., ΔLST), which is likely not sensitive to elevation.

For each pair of selected cells within a window, we calculated “standardized” forest change (ΔF) as:

$$\Delta F=\Delta F_{foc}-\Delta F_{ref}$$

where ΔF_foc_ is the one-decadal change in forest cover (cover in 2010 minus cover in 2000) in the focal cell, and ΔF_ref_ is the one-decadal change in forest cover in the reference cell (Fig 1). Positive ΔF values are indicative of forest gain (i.e., forestation) in the focal cell relative to the reference cell, and negative values are indicative of forest loss (i.e., deforestation). We used the same approach to calculate standardized changes in climatic variables (ΔLST, ΔET and Δalbedo), extracting the values for each variable from the exact same focal and reference cells chosen during the forest change window searching strategy (S5 Fig). By performing temporal comparisons (i.e. analysis of one-decade changes) through the window searching strategy, and focusing our subsequent analysis on these standardized values, we were able to minimize differences in background (regional) climate between each pair of cells, thus performing a more direct test of how forest change affects local climate.

### Analysis of global impacts of forest change on local climate

We obtained a total of 36,493 valid pairs of annual ΔLST_foc_ and ΔLST_ref_ values, 14,869 of Δalbedo_foc_ and Δalbedo_ref_, and 97,618 of ΔET_foc_ and ΔET_ref_ (see S5 and S6 Figs). The number of pairs varied among variables due to the quality control adopted for each variable, as explained above. To evaluate whether the values of each variable differed between focal and reference cells, we performed separate tests for each region (tropical = 20°S—20°N; temperate = 20°S—50°S and 20°N—50°N, and boreal = > 50°S and > 50°N, as in [15]), and for each forest change category (deforestation or forestation; see S1 Table). Tests were performed using mixed-effect models, with the type of cell (reference or focal) as a fixed effect, and the searching window as a random effect. This modeling approach is equivalent to a paired t-test, allowing comparison of each pair of focal-reference cells within each window. In addition, the mixed-modeling approach helps to control parameter overestimation due to spatial autocorrelation [31]. To determine the most suitable autocorrelation structure (which is unknown a priori), we built alternative models with the same fixed and random effects (as above), but with different correlation structures: spherical (corSpher), linear (corLin), rational quadratic (corRatio), gaussian (corGaus), and exponential (corExp) [31]. Models included correlation structure with the form = ~ longitude + latitude, as implemented in the R package “nlme” [32]. A model with no correlation structure was also considered for comparison [31]. We compared models via model selection based on AICc [33], using the R package “MuMIn” [34]. In all comparisons, models with some type of autocorrelation structure were always more plausible than the model with no structure (S1 Table). We used the top-ranked model in each model selection to estimate mean effects, standard errors and confidence intervals (S1 Table).

For comparative purposes, we also analyzed ΔLST values considering daytime and nighttime LST, separately (S1D and S1E Table; S7 and S8 Fig). In addition, to assess whether similar amounts of deforestation and forestation had impacts of similar magnitude on LST, we fit the mixed-effect models only to pairs of cells with standardized values of absolute forest change around 50% (i.e., |40%| < ΔF < |60%|). Within this 40–60% range, the distribution of forest change values was similar for forestation and deforestation (S9 Fig), thus allowing direct comparisons between the two types of forest change. We fit these models separately for each forest change category (deforestation or forestation) and region (tropical, temperate or boreal), and obtained the mean ± 95% CI estimates from the top-ranked models.

**Fig 2:**
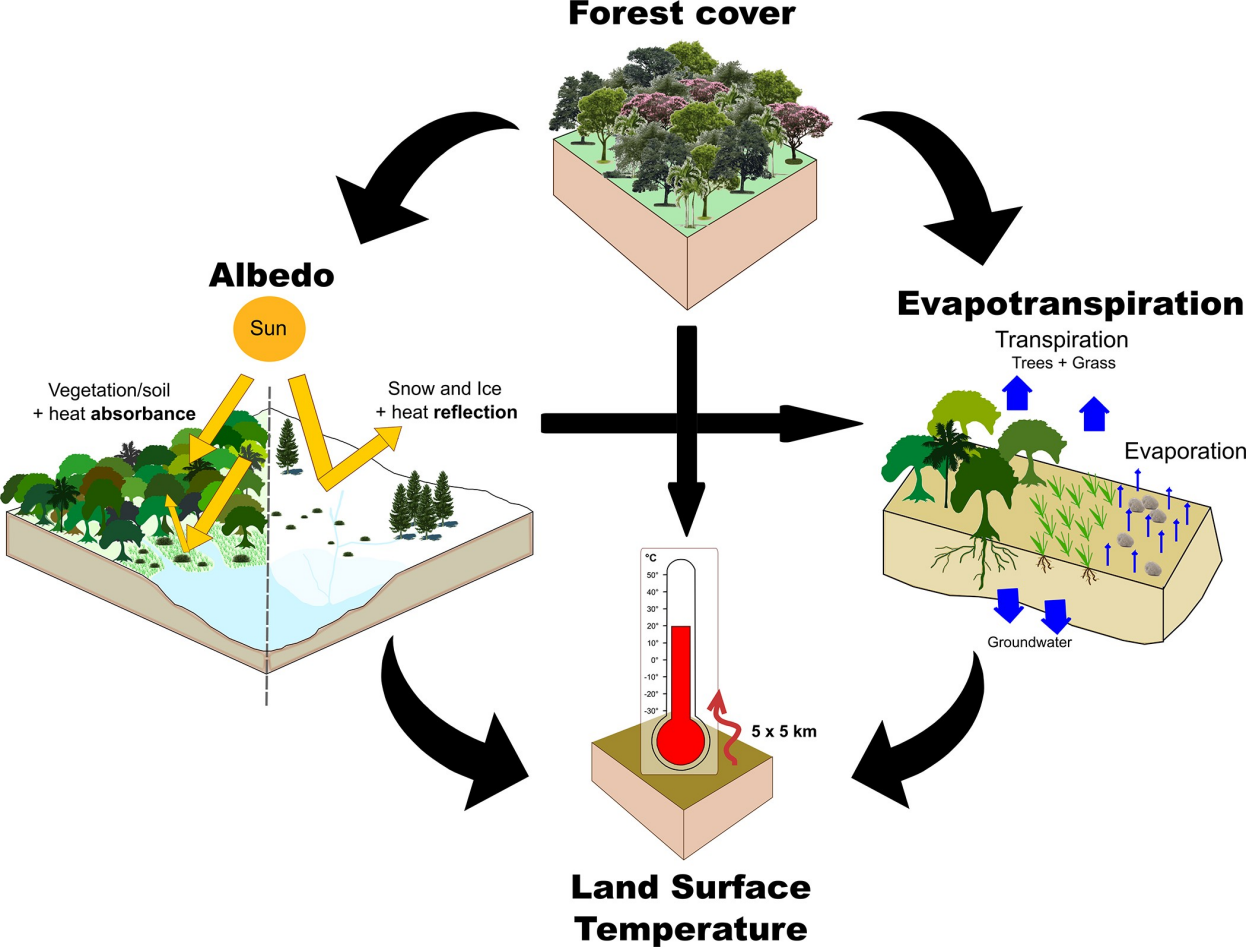
Scheme depicting presumed relationships between forest cover and climatic variables (albedo and evapotranspiration). These climatic variables are regarded as the main drivers of land surface temperature change. Heat is more readily absorbed by vegetation, soil and water bodies (lower albedo values), than surfaces with snow and ice (higher albedo values). By estimating condensed water from substrate evaporation and vegetation transpiration, we quantify the available water in the air (evapotranspiration). At the same time, heat absorbance induces greater water condensation by both soil and vegetation.

We used multilevel structural equation modeling [22] to (i) quantify causal relationships among ΔF, Δalbedo, ΔET, and ΔLST, and (ii) assess how these relationships vary across regions (tropical, temperate, and boreal). Multilevel equation models are an extension of traditional linear path models, but allow incorporation of spatial autocorrelation [22,35]. For this analysis, we used all 8,419 pairs of cells with information on all variables (S5D Fig; N = 409, 7794 and 216, for tropical, temperate, and boreal regions, respectively). We first built a general path model that summarizes the most likely relationships among variables, as currently considered in the literature (e.g. [6,15,16]; Fig 2). As the purpose of the model was to facilitate understanding, more than to fully explain patterns, we ignored other potentially important variables, such as cloud formation and precipitation, which may also be affected by forest change and may affect LST [17]. Thus, this simple model had only three response variables, which were assumed as dependent on one or more variables, as follows: (i) ΔLST depending on ΔF, Δalbedo, and ΔET; (ii) Δalbedo depending on ΔF; (iii) ΔET depending on ΔF and Δalbedo (Fig 2).

We fit each response variable to a generalized least squares linear model (gls) using the function “gls” from the R package “nlme” [32]. To control spatial autocorrelation, the three individual models (above) included a rational quadratic spatial correlation structure (corRatio), with the form = ~ longitude + latitude as implemented in the R package “nlme” [32]. This correlation structure was chosen since it was the top-ranked in most tests analyzing the relationship between ΔF and ΔLST, Δalbedo, or ΔET (S1 Table). We fit the general path model for each region (tropical, temperate, and boreal) separately, using the R package “piecewiseSEM” [35]. We then recorded the standardized path coefficients for each link and compared the coefficients across regions (see Fig 2). The coefficients were robust to variation in the correlation structure used (S2 Table), and similar for models with the three most plausible structures based on AICc (“corRatio”, “corExp”, and “corGaus”, see S1 Table).

We also tested the fit of simpler path models to the data, i.e., models without one or more of the causal links that are present in the general model (Fig 2). However, these simpler models had poor global fits to the data for the boreal and temperate regions, as inferred from their significant Fishers’ C-statistics (p < 0.05), indicating the missing links were important [22,35]. Thus, we kept all links and only used the general model to estimate the path coefficients, as explained above. Due to the absence of missing links in this model, it was not possible to test its global fit to the data [22,35]. Therefore, we assessed only the local fit of the three individual gls models, using model residuals and R^2^ values, which indicated a good fit of the model to the data for all regions.

### Modelling future changes in local climate in Brazil

To show how our approach can be further applied, we also used a multilevel equation model to explore how future (2010–2050) forest change may affect local LST. We analyzed forest cover and climate data (LST, albedo, and ET) for Brazil from 2000–2010 and 2001–2011, respectively, using the same MODIS-derived data and previously explained procedures. Our choice of Brazil as a model was based on both data availability, and the large spatio-temporal variation in forest cover within Brazil, which both has two highly threatened biodiversity hotspots (Atlantic Forest and Cerrado) and the largest tropical forest in the planet (Amazon). We also applied the window-searching algorithm for continental Brazil and obtained 1,953 valid pairs of focal-reference cells for analysis. For each pair, we quantified ΔF, ΔLST, Δalbedo and ΔET, and then used multilevel path analysis to quantify the total effects (direct + indirect) of ΔF on ΔLST. We fit the general path model with a rational quadratic spatial correlation structure (corRatio; see Data Analysis) and recorded all standardized path coefficients. The direct effects of ΔF on ΔLST corresponded to the coefficients of the single path linking the two variables (see Fig 2). Three indirect effects were also calculated: (i) ΔF → Δalbedo → ΔLST; (ii) ΔF → ΔET → ΔLST; and (iii) ΔF → Δalbedo → ΔET → ΔLST. Each indirect effect was calculated by multiplying the standardized path coefficients along the pathway. Finally, we obtained the total effect of ΔF on ΔLST by summing the direct effect and the three indirect effects.

We estimated future forest change in Brazil (2010–2050) using data from the Reducing Emissions from Deforestation and Forest Degradation project [23]. These data were generated by combining information from multiple sources on several variables, including vegetation, protected areas, transportation costs, and planted forest values (see [23]). We analyzed data from two scenarios: “business-as-usual” (BAU) and “forest code” (FC). The BAU scenario assumes no effective control of deforestation in Brazil, allowing illegal deforestation in all biomes, except in the Atlantic Forest, which has specific additional legislation (Atlantic Forest Law 11428/2006). According to the BAU scenario, deforestation will occur across 606,000 km^2^, with zero additional forestation [23], over the 2010–2050 period. The BAU scenario functions as a “control” scenario to evaluate the effects of the implementation of the Brazilian Forest Code, a national law passed in 1965 intended to reduce illegal deforestation, which was most recently modified in 2012 (Law 12651/2012). On the other hand, the FC scenario assumes no illegal deforestation, as well as full compliance with the terms of the Forest Code, including forest restoration in legal reserves, minimum quotas for forest reserves number and size, amnesty for previous deforestation on small farms, and the enforcement of the Atlantic Forest Law. According to the FC scenario, deforestation will occur across 79,000 km^2^, with additional forestation on 110,000 km^2^ of previously illegally deforested land [23].

For each scenario, we calculated forest cover in 2010 and 2050, and then forest change (2050 minus 2010), for each 5’ x 5’ cell. We downscaled forest change values to 0.05° to match the MODIS LST data, using the bilinear interpolation method from the R package “raster” [36]. We then used the forest change values and the standardized coefficients of the path model to predict ΔLST for each 0.05 x 0.05° cell across Brazil. This analysis assumes that the current relationships between forest change, albedo, ET and LST will remain unchanged. Although unlikely, this assumption allows us to understand how different scenarios of local forest change may impact LST, independent of their feedbacks with regional climate, which are much more uncertain to predict.

To compare our predictions of local LST change with the regional climate changes predicted for Brazil, we analyzed the CMIP5 climate projections (IPCC 5th Assessment; available at www.worldclim.org/version2). For comparison, we chose two representative concentration pathways (RCP4.5 and RCP8.5) from the global climate model MIROC5 (Model for Interdisciplinary Research on Climate, Version 5), to obtain the average annual surface temperature for 2050 (average for years 2041–2060) with 30 arc-seconds resolution. RCPs describe different pathways of anthropogenic greenhouse gas emissions, based on variables such as human population size, energy use, land use, and climate policy [37]. RCP 4.5 is an intermediate scenario, predicting annual greenhouse gas emissions from 530–580 GtCO2 in 2050, whereas RCP 8.5 assumes very high emissions (>1000 GtCO2). We quantified the predicted change in the mean annual surface temperature as the difference between future temperature (2050) and current temperature (average from 1970 to 2000; [36]). Finally, we calculated the average ± SE change both across Brazil and across the tropical region (0–20° N or S).

## Results

### Global impacts of forest change on local climate

**Fig 3:**
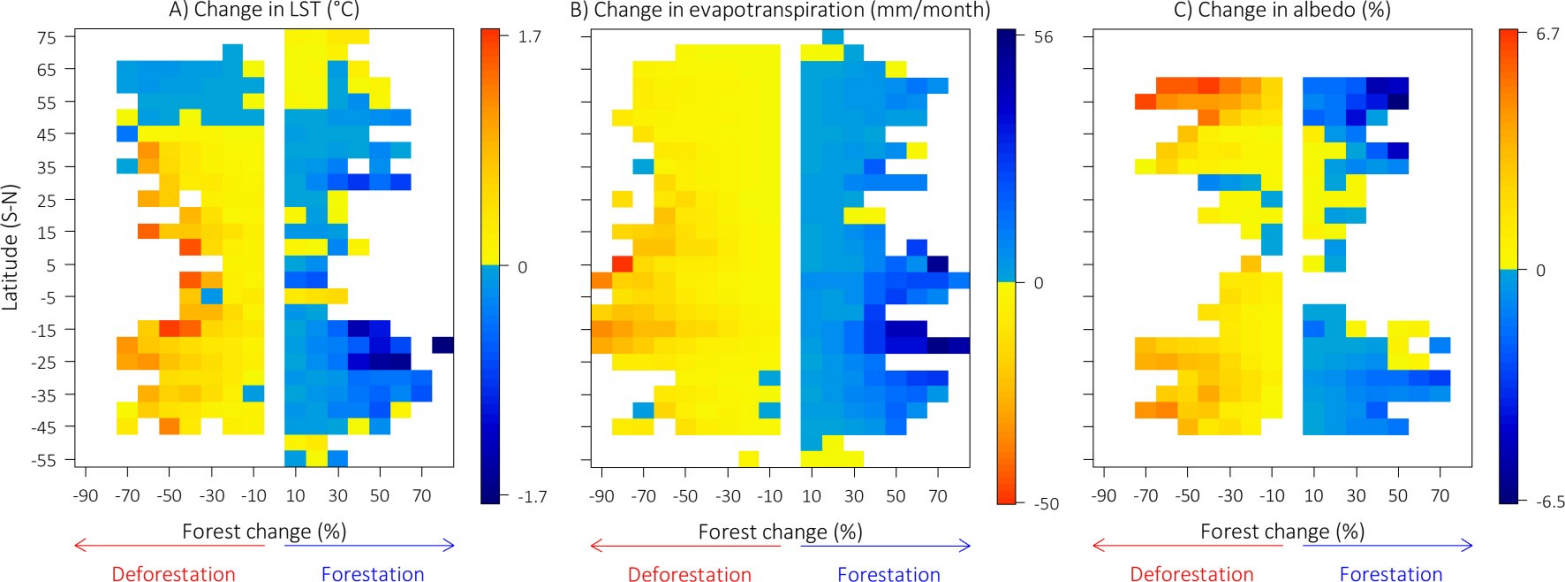
Effects of forest change on local climate change. (A) Change in annual land surface temperature (LST). (B) Change in evapotranspiration. (C) Change in albedo. Each cell in the plots represents decadal changes in annual means of climatic variables (2011–2001) following decadal forest changes (2010–2000), calculated originally for 0.05 x 0.05° cells, and grouped into bins of 5° latitude and 10% forest change to facilitate visualization. Note that the number of bins with good-quality data is higher for ET than for albedo.

Globally, deforestation and forestation had opposite effects on local climate, and these effects varied with latitude (Fig 3). Deforestation led to consistent warming of 0.38 ± 0.02°C (mean ± SE) in tropical regions and 0.16 ± 0.01°C in temperate regions, but in boreal regions led to slight cooling of -0.04 ± 0.003°C (Fig 3A; for full statistics, see S1 Table). Conversely, forestation had cooling effects in tropical (-0.18 ± 0.02°C) and temperate regions (-0.19 ± 0.02°C), but null effects in boreal regions (0.01 ± 0.01°C). Forest change had opposite effects on daytime and nighttime LST (S7 Fig), and the magnitude of change was greater for daytime compared to both nighttime or annual LST (compare Fig 3 and S7 Fig).

**Fig 4:**
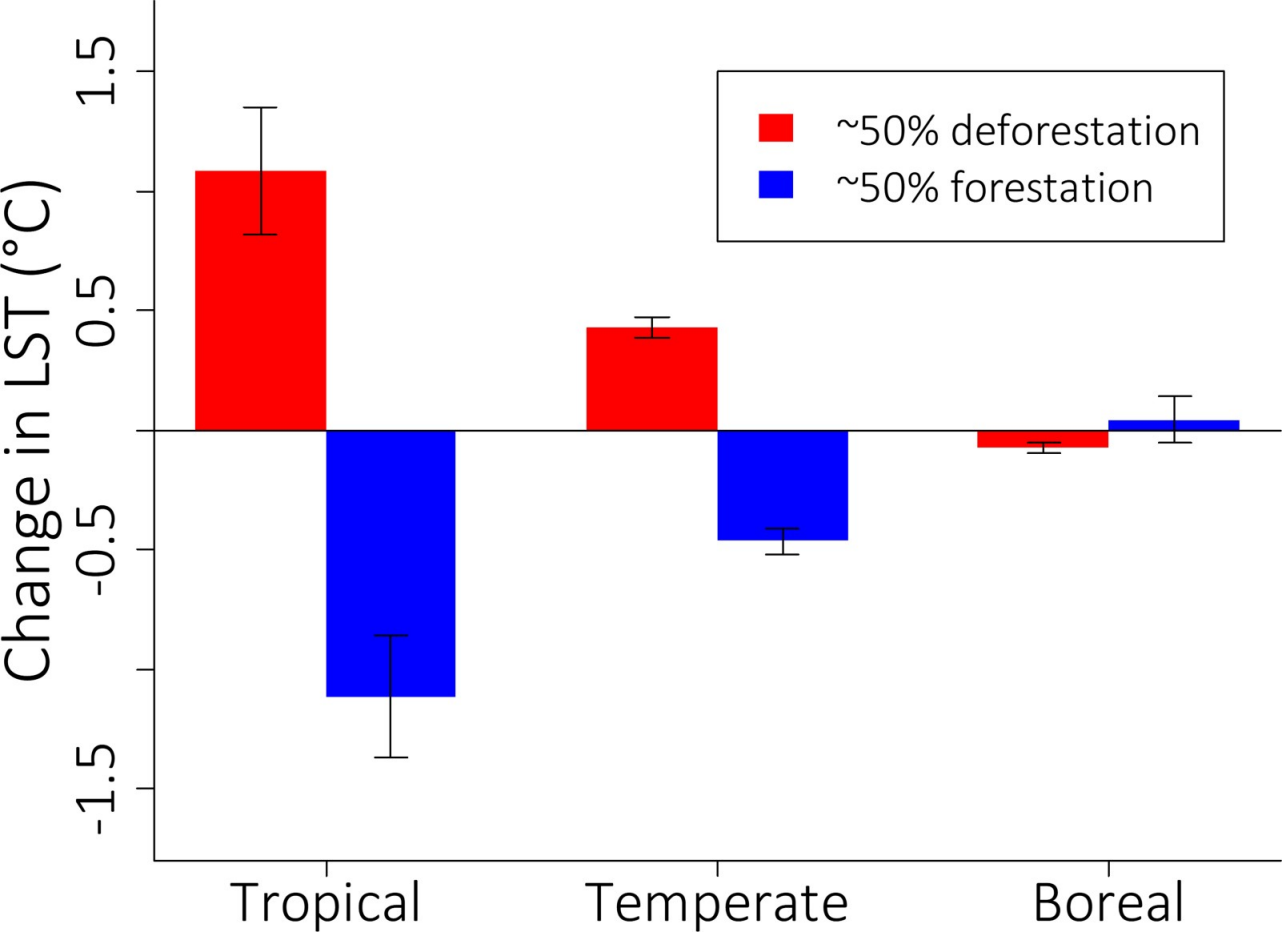
Comparative effects of deforestation (red) and forestation (blue) on annual land surface temperature (LST) change. This analysis considered only cells with ~50% (40–60%) of decadal forest change (2010–2000). Positive (negative) values indicate a warming (cooling) effect of forest change. Bars indicate averages and 95% confidence intervals. The number of cell pairs analyzed (left to right) was 40, 15, 694, 268, 861 and 25.

Similar rates (estimated at a value of ~50%) of deforestation and forestation had impacts of similar magnitude on annual LST, but in opposite directions regardless of the region under consideration (Fig 4). For example, deforestation of ~50% in tropical regions led to strong warming of 1.08 ± 0.25°C, while forestation of ~50% led to strong cooling of -1.11 ± 0.26°C. A similar pattern also occurred for the temperate region, though with lower magnitudes. In contrast, the boreal region demonstrated an effect on annual LST both lower in magnitude and in opposite direction (Fig 4).

**Fig 5:**
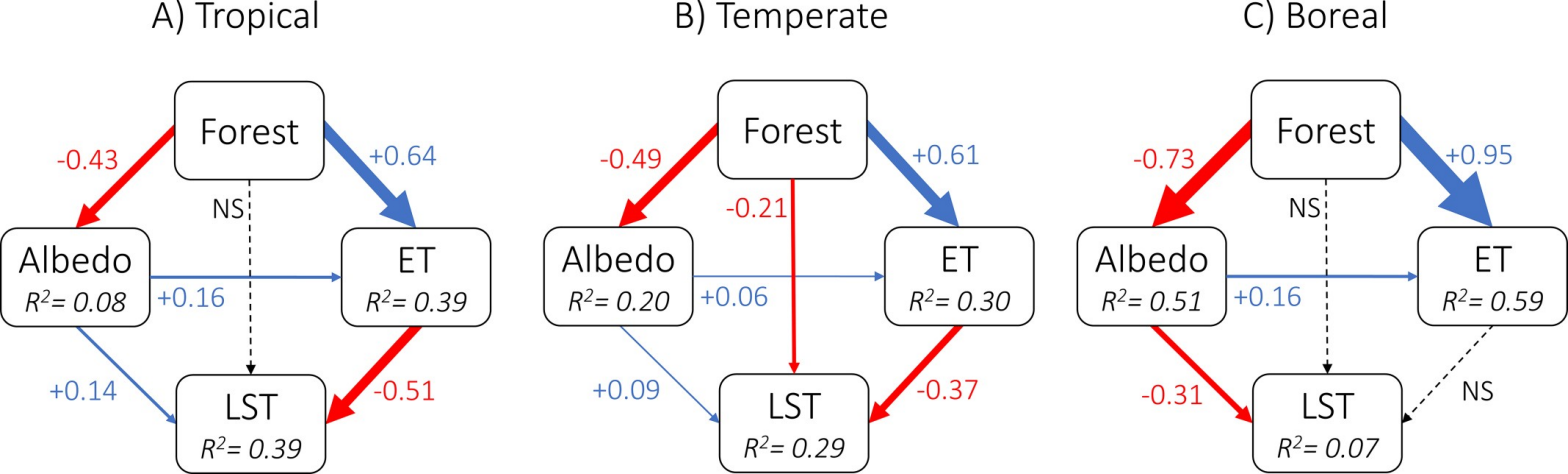
Path diagrams showing the direct and indirect effects of forest change on annual land surface temperature (LST). Indirect effects are assumed to be caused by changes in albedo and evapotranspiration (ET). Arrows are scaled in proportion to the absolute standardized path coefficients (numbers). Red and blue arrows indicate negative and positive coefficients, respectively. Coefficients for non-significant paths (p > 0.05) are not shown (NS). R^2^ values indicate the coefficient of determination of the component models for each response variable.

Changes in annual LST were correlated to changes in albedo and ET (Fig 5). A structural equation model including only these two variables explained 29% of the variation in LST across the globe. The explanatory power of this model was much higher for tropical (39%) than for boreal regions (7%; Fig 5). Forestation increased ET (positive coefficients in Fig 5), which then reduced LST in all regions (negative coefficients), but especially in tropical and temperate areas (Fig 5). In boreal areas, the effect of ET on annual LST was negligible. Forestation also reduced albedo in all regions (negative coefficients in Fig 5), especially in boreal areas (Fig 5C).

### Future changes in local climate in Brazil

The total effect of ΔF on ΔLST in Brazil was estimated as -0.64, meaning that, on average, an increase in one standard deviation of forest cover (= 21.7%) reduced LST in 0.64 standard deviations, i.e., 0.36°C (0.64 x 0.57° C, 0.57° C being the standard deviation of ΔLST). Combined indirect effects of ΔF on ΔLST (-0.41) were almost twice as strong as the direct effect (-0.23). Similar estimates were obtained using alternative correlation structures (corExp: indirect = -0.42, direct = -0.22; corGaus: indirect = -0.38, direct = -0.28). The model fit the data well, as judged by the component models, which explained a total of 43%, 28%, and 54% of the variation in ΔLST, Δalbedo and ΔET, respectively.

**Fig 6:**
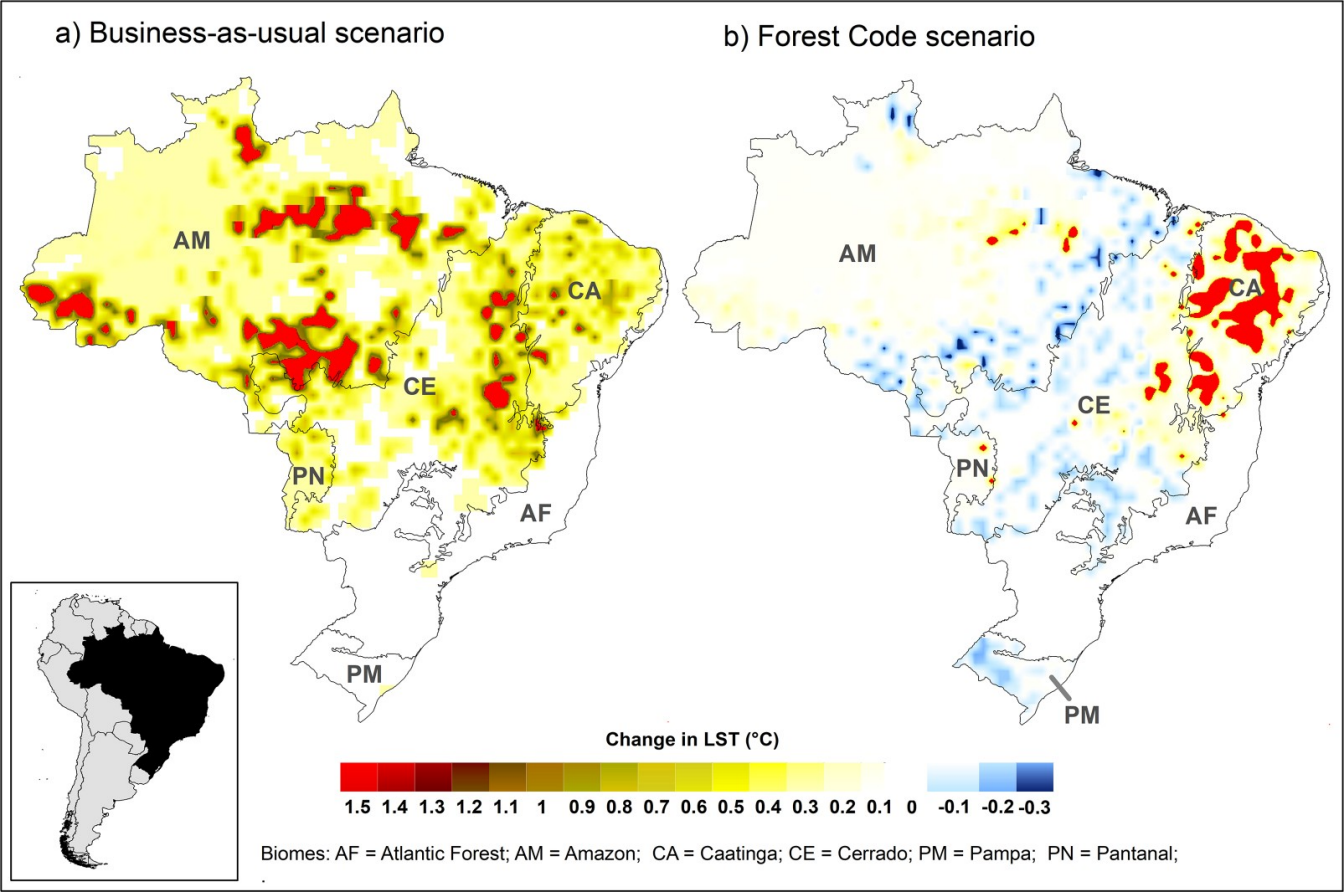
Predicted future changes in annual land surface temperature (LST) for Brazil from 2010 to 2050. Analyses were based on changing forest cover predicted by two land use change scenarios: (a) Business-as-usual (BAU) scenario, in which land use follows the same pattern observed in 2000; (b) Forest Code (FC) scenario, in which the 2012 Brazilian environmental legislation (“Forest Code”) is fully implemented. Brazilian biomes: AM = Amazon; CE = Cerrado; CA = Caatinga; AF = Atlantic Forest; PM = Pampa; PN = Pantanal.

In the BAU scenario, extensive deforestation was predicted to increase annual LST up to 1.45°C (average = 0.11, ranging from 0 to 1.45°C), especially in the Amazon and Cerrado domains (Fig 6A). As no forestation is predicted from the BAU scenario, no cooling from positive forest change was predicted. On the other hand, LST would be much less affected under the FC scenario (0.02°C on average, ranging from -0.33 to 1.29°C; Fig 6B). Under this scenario, clear increases in LST are expected only for the Caatinga domain in northeastern Brazil (Fig 6B). In addition, under the FC scenario, forestation may lead to local cooling, especially in the Amazon and Cerrado (Fig 6B). These estimates of local warming under both land use scenarios are lower than the regional warming predicted from global change for Brazil under both the optimistic scenario (RCP 4.5; average ± SE: 1.99 ± 0.0002°C) and the more pessimistic scenario (RCP 8.5; 2.4 ± 0.0002°C).

## Discussion

Forestation and deforestation were pervasive across large areas of the globe from 2000 to 2010 [5]. Here we show that these widespread processes had strong and opposite effects on local LST, albedo and ET worldwide, confirming and expanding the findings of recent studies [10–12]. In particular, we show that forestation (both passive and active) has the potential to reverse the effects of deforestation on LST, albedo and ET. This finding provides further evidence of the need to reduce deforestation and promote forest recovery—especially in tropical and temperate regions [5,12]. We found that the effects of forest change on LST are largely mediated by changes in albedo and ET. Finally, we show that current land use policies are very likely to impact future local climate, as exemplified by our case study in Brazil.

The climatic effects of deforestation and forestation mirrored one another and were stronger with increasing levels of either process, as also documented in two recent studies [10,11]. In tropical regions, impacts of deforestation on local climate were higher than that of forestation (0.38°C versus -0.18°C, respectively), because forest loss was more expansive than forest gain [5; see also S6 Fig]. However, when we compared identical amounts of forestation and deforestation, their climate impacts were similar in magnitude (Fig 4). The apparently minor effects of forest change on average annual LST in boreal regions may reflect the complex seasonal dynamics of vegetation-albedo effects in these regions, through absorbed shortwave radiation and surface roughness [9,15,17,18]. Similar effects of forest losses and gains have also been detected for surface air temperatures in a recent semi-empirical analysis [10], and also for potential and actual LST changes [11]. In addition to deforestation’s effects on LST, our analyses importantly reveal that deforestation consistently decreases ET and increases albedo in all regions, while forestation has the opposite effects (Fig 4B and 4C). Together, these results provide additional strong evidence that forestation and deforestation have pervasive and opposite effects on local climate worldwide [10,11]. Importantly, the climatic effects of changing forest cover were stronger for daytime compared to nighttime LST, and may be even more pronounced at ground level (i.e. beneath the canopy), with potentially pervasive direct impacts on many organisms [38–40].

While the effectiveness of tree planting to mitigate global warming is still under debate [16,17,41], our results suggest that the local climatic effects of deforestation can be reversed with forestation, even over short time periods (< 10 yr.), as also recently suggested [10,11]. A large portion of the forestation observed from 2000 to 2010 corresponded to tree planting for commercial purposes [4,5], with an additional contribution of active restoration efforts and passive regeneration of native forests [42,43]. Unfortunately, the forest cover maps of Hansen *et al*. [5] do not differentiate between native and planted forests, which have different impacts for biodiversity and for the provision of ecosystem services, for example as discussed in Tropek et al. [44]. This limitation may have contributed to some of the unexplained variability in our results (see color pattern in Fig 3 and R^2^ values in Fig 5). Despite this limitation, however, the high-resolution maps were suitable for understanding how forest cover *per se* (regardless of native or planted origin) affects local climate, as they adequately capture the distribution of biophysical features of interest (tree cover) across the Earth’s surface [5]. Overall, our analyses, combined with those performed recently in two studies [10,11], show that both passive and active forestation have the potential to reverse the climatic effects of deforestation.

Our structural equation model had higher explanatory power for tropical (39%) than for boreal regions (7%), likely due to the more complex seasonal dynamics of vegetation-albedo effects in boreal regions [11,15,17,18]. In boreal areas, the effect of ET on LST was negligible, reflecting the overall low amount of ET in these areas due to the relatively dry and cold climate [11,18]. In boreal regions, reduced albedo promoted by forestation resulted in a slight warming effect (Fig 5C), due to the higher absorption of shortwave radiation during the day and small latent heat release [18], increasing nighttime temperatures (see S7 Fig). The low-to-moderate explanatory power of the structural equation model, even for the tropical region, is likely to reflect the heterogeneity of the forest cover dataset (as discussed above), as well as the seasonal and daily variation in local climate. Despite these limitations, the structural equation model provides additional evidence that the global effects of forest change on LST are modulated by albedo and ET effects, which vary latitudinally [6,11,13–16].

Our study case of Brazil illustrates that current land use policies can impact future local climate. Importantly, we found that a business-as-usual scenario—associated with extensive deforestation—could substantially increase annual LST, especially in the Amazon and Cerrado biomes. Predicted deforestation in the Amazon is typically concentrated near roads and rivers [45], and in the “Arc of Deforestation” along the biome’s southern and eastern boundaries [46]. On the other hand, under the Forest Code scenario, we found that clear increases in LST are only expected for the Caatinga, which is likely to be deforested due to cropland expansion regardless of enforcement of the Forest Code [23]. Our models for Brazil are not intended to predict future climate, but rather to provide scenario-based examples of how future forest changes may affect LST. Our approach may be included in more complex and predictive models, which must consider variation in precipitation and overall background climate to generate reasonable predictions of future LST.

Local vegetation-driven changes in climate are particularly important in the context of global climate change because they influence both regional and global circulation and precipitation patterns [47–49], and may have a compounding influence on observed local temperature increases. In tropical regions, for example, the 1.08 ± 0.25°C local warming caused by a 50% reduction in forest cover may add to the estimated 1.7 ± 0.0001°C regional warming expected from global change under an intermediate and realistic warming scenario (MIROC5 model, RCP 4.5). Increased temperatures in already hot locations may increase human mortality rates and electricity demands, reduce agricultural yields and water resources, and contribute to biodiversity collapse, particularly in tropical regions [50–55]. Furthermore, local warming may cause shifts in species distributions [56], including for species involved in infectious disease transmissions [57]. Because forest change lies at the heart of the warming problem, initiatives to reduce deforestation should remain a priority. In conjunction, both passive (natural) and active (planted) forestation should be promoted within originally forested areas in tropical and temperate biomes as restored forest has the potential to benefit biodiversity [58] and also reverse the climatic effects of deforestation.

## Supporting information

S1 Fig Results for 2000–2010 climatic data. a Effects of decadal (2010–2000) forest change on decadal changes in annual land surface temperature (LST), evapotranspiration (ET) and albedo. b Comparative effects of deforestation (red) and forestation (blue) on LST changes across regions, considering only cells with ~50% of forest change. c Path diagrams showing the direct and indirect effects of forest change on LST.
(DOCX)

S2 Fig Effects of forest change on albedo change considering the albedo GLASS (Global LAnd Surface Satellites) dataset. Each cell in the plots represents decadal (2011–2001) changes in annual means of albedo calculated for 0.05 x 0.05° cells grouped into bins of 5° latitude and 10% forest change. For this analysis, we first calculated monthly averages, and then annual averages, considering only pixels that had good-quality information for all months in a year, i.e., flags “00” and “01” (indicating uncertainty < 5 and < 10%, respectively). In some months, pixels above 55°N had only uncertain estimates (flag “11”) and thus were not used in the analysis.
(DOCX)

S3 Fig Results for searching windows of 9 x 5 cells (longitude x latitude). a Effects of decadal (2010–2000) forest change on decadal (2011–2001) changes in annual land surface temperature (LST), evapotranspiration (ET) and albedo. b Comparative effects of deforestation (red) and forestation (blue) on LST changes across regions, considering only cells with ~50% of forest change. c Path diagrams showing the direct and indirect effects of forest change on LST.
(DOCX)

S4 Fig Results for a different classification of focal and reference cells. For this analysis, we classified “focal” cell as cells with absolute forest cover change > 10%, and “reference” cells as cells with absolute forest cover change < 2% a Effects of decadal (2010–2000) forest change on decadal (2011–2001) changes in annual land surface temperature (LST), evapotranspiration (ET) and albedo. b Comparative effects of deforestation (red) and forestation (blue) on LST changes across regions, considering only cells with ~50% of forest change. c Path diagrams showing the direct and indirect effects of forest change on LST.
(DOCX)

S5 Fig Spatial distribution of all valid pairs of focal/reference cells used in the analyses. Each pair is represented as a single red point. A) Pairs used in the analyses of annual land surface temperature (LST; N = 36,493). B) Pairs used in the analyses of evapotranspiration (ET; N = 97,618). C) Pairs used in the analyses of albedo (N = 14,869). D) Pairs used in the path analyses, which contained valid information for the three climatic variables of interest (annual LST, ET and albedo; N = 8,419).
(DOCX)

S6 Fig Histograms of standardized change values used in the analyses. Four variables are shown: forest cover (first column), annual land surface temperature (LST, second column), evapotranspiration (ET, third column) and albedo (fourth column). Values for each variable were recorded for each pair of focal/reference cells, as the one-decade change observed in the focal cell minus the one-decade change in the reference cell (2010–2000 for forest cover, 2011–2001 for climatic variables). Values are shown separately for each region (Tropical, Temperate and Boreal) and for all regions combined (World). The blue vertical line indicates no difference between the focal and the reference cell in their one-decade change value (standardized change value = 0).
(DOCX)

S7 Fig **Effects of forest change on daytime (a) and nighttime (b) temperature.** Each cell in the plots represents decadal (2011–2001) changes in annual means of climatic variables calculated for 0.05 x 0.05° cells grouped into bins of 5° latitude and 10% forest change. Positive (negative) values indicate a warming (cooling) effect of forest change.
(DOCX)

S8 Fig **Histograms of daytime (left) and nighttime (right) land surface temperature (LST) change values.** Values were recorded for each pair of focal/reference cells, as the one-decade (2010–2000) change observed in the focal cell minus the one-decade change in the reference cell. Values are shown separately for each region (Tropical, Temperate and Boreal) and for all regions combined (World). The blue vertical line indicates no difference between the focal and the reference cell in their one-decade change value (standardized change value = 0).
(DOCX)

S9 Fig **Histograms of standardized forest change values for deforestation (A) and forestation (B).** These values were used in the analyses comparing effects of deforestation and forestation on annual land surface temperature change (see Fig 4 in the main text).
(DOCX)

S1 Table Summary of tests evaluating the effects of forest change on local climate. Models were fit separately for each climatic variable (a—e), for each region (tropical, temperate or boreal*), and for each category of forest change (deforestation or forestation**). All models included the type of cell (reference or focal) as a fixed effect, and the searching window as a random effect. To account for spatial autocorrelation, different correlation structures were tested for each model, and the most suitable correlation structure was determined via model selection. Average, 95% confidence intervals and p-values were obtained from the top-ranked model only. N = number of focal/reference pairs of cells; t = t-statistic; p = significance level. Tests with p ≤ 0.05 are highlighted in bold. *Tropical = 20°S—20°N; Temperate = 20°S—50°S and 20°N—50°N; Boreal = >50°S and >50°N. **Deforestation and forestation correspond to decreases and increases in forest cover > 15%, respectively, from 2000 to 2010. ***Correlation structure of the top-ranked model. The correlation structures tested were corSpher, corLin, corRatio, corGaus,and corExp, with form = ~ longitude + latitude as implemented in the R package “nlme” (51).
(DOCX)

S2 Table Standardized coefficient estimates for multi-level structural equation models. Coefficients are presented for each link of the path model (“Response”—“Predictor” pair). Models were fit separately for tropical, temperate and boreal regions. Three alternative spatial correlation structures were tested: rational quadratic (corRatio), exponential (corExp) and gaussian (corGaus). LST = change in annual land surface temperature; ET = change in evapotranspiration; Forest = change in forest; Albedo = change in albedo. NS = statistically non-significant coefficients (p > 0.05). See also Fig 4 in the main text for a graphical presentation of the coefficients of the model with corRatio structure.
(DOCX)
