# Supplementary material for: Impacts of forestation and deforestation on local temperature across the globe
Source: PLoS One. 2019 Mar 20;14(3):e0213368. doi: 10.1371/journal.pone.0213368 (PMC6426338; doi:10.1371/journal.pone.0213368)
Supplement: S2 Fig — Each cell in the plots represents decadal (2011–2001) changes in annual means of albedo calculated for 0.05 x 0.05° cells grouped into bins of 5° latitude and 10% forest change. For this analysis, we first calculated monthly averages, and then annual averages, considering only pixels that had good-quality information for all months in a year, i.e., flags “00” and “01” (indicating uncertainty < 5 and < 10%, respectively). In some months, pixels above 55°N had only uncertain estimates (flag “11”) and thus were not used in the analysis. (DOCX) [file pone.0213368.s002.docx]

S2 Fig. Effects of forest change on albedo change considering the albedo GLASS (Global LAnd Surface Satellites) dataset. Each cell in the plots represents decadal (2011 – 2001) changes in annual means of albedo calculated for 0.05 x 0.05º cells grouped into bins of 5º latitude and 10% forest change. For this analysis, we first calculated monthly averages, and then annual averages, considering only pixels that had good-quality information for all months in a year, i.e., flags “00” and “01” (indicating uncertainty < 5 and < 10%, respectively). In some months, pixels above 55ºN had only uncertain estimates (flag “11”) and thus were not used in the analysis.
