# Supplementary material for: Impacts of forestation and deforestation on local temperature across the globe
Source: PLoS One. 2019 Mar 20;14(3):e0213368. doi: 10.1371/journal.pone.0213368 (PMC6426338; doi:10.1371/journal.pone.0213368)
Supplement: S3 Fig — a Effects of decadal (2010–2000) forest change on decadal (2011–2001) changes in annual land surface temperature (LST), evapotranspiration (ET) and albedo. b Comparative effects of deforestation (red) and forestation (blue) on LST changes across regions, considering only cells with ~50% of forest change. c Path diagrams showing the direct and indirect effects of forest change on LST. (DOCX) [file pone.0213368.s003.docx]

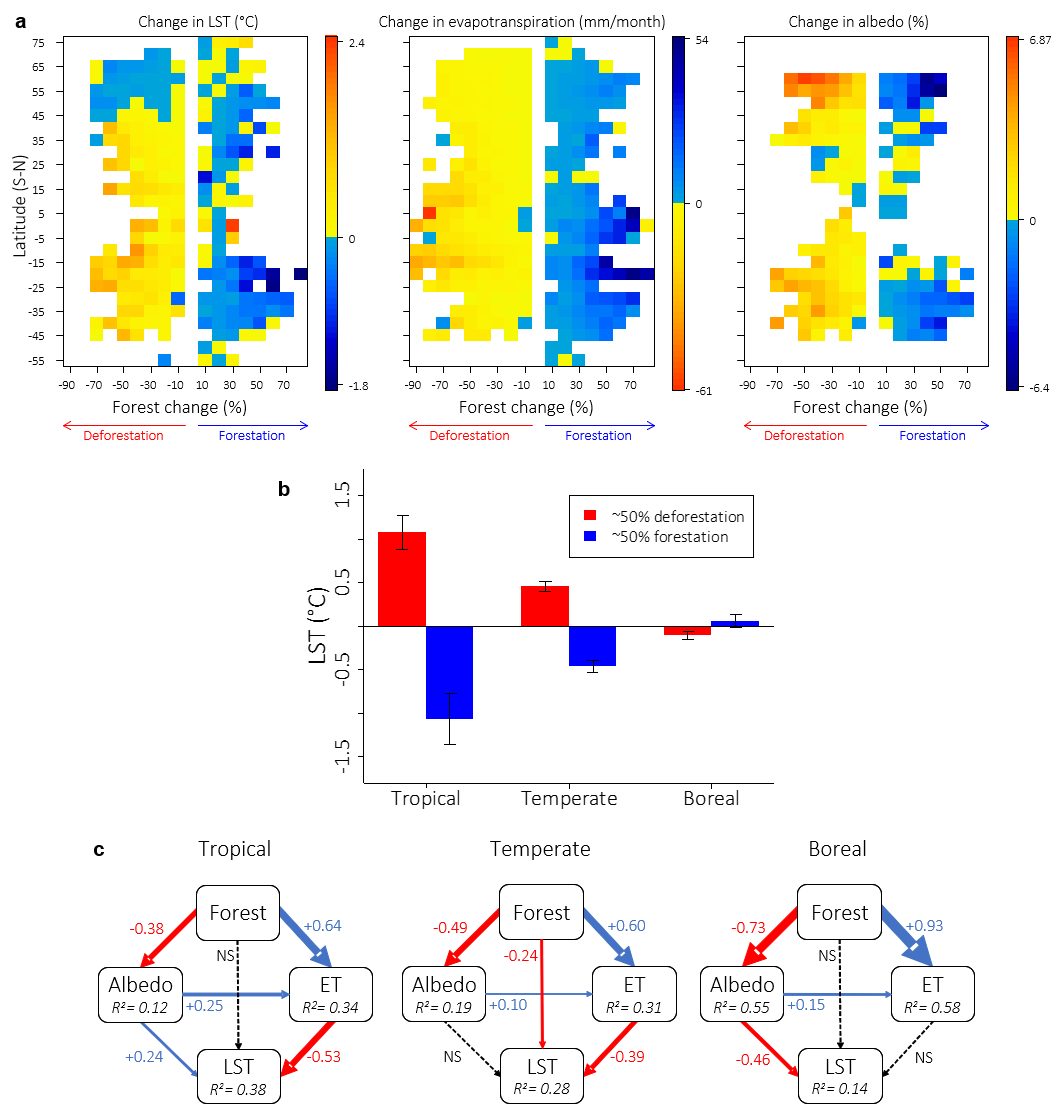


**S3 Fig. Results for searching windows of 9 x 5 cells (longitude x latitude).** a Effects of decadal (2010 - 2000) forest change on decadal (2011 – 2001) changes in annual land surface temperature (LST), evapotranspiration (ET) and albedo. b Comparative effects of deforestation (red) and forestation (blue) on LST changes across regions, considering only cells with ~50% of forest change. c Path diagrams showing the direct and indirect effects of forest change on LST.
