# Supplementary material for: Impacts of forestation and deforestation on local temperature across the globe
Source: PLoS One. 2019 Mar 20;14(3):e0213368. doi: 10.1371/journal.pone.0213368 (PMC6426338; doi:10.1371/journal.pone.0213368)
Supplement: S5 Fig — Each pair is represented as a single red point. A) Pairs used in the analyses of annual land surface temperature (LST; N = 36,493). B) Pairs used in the analyses of evapotranspiration (ET; N = 97,618). C) Pairs used in the analyses of albedo (N = 14,869). D) Pairs used in the path analyses, which contained valid information for the three climatic variables of interest (annual LST, ET and albedo; N = 8,419). (DOCX) [file pone.0213368.s005.docx]

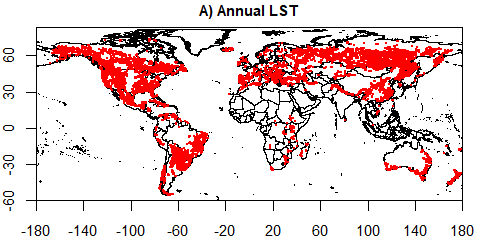


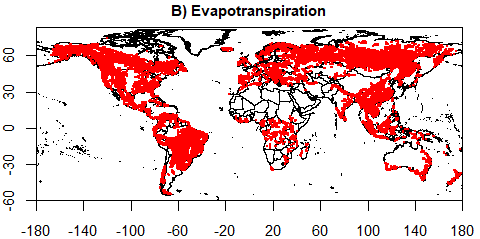


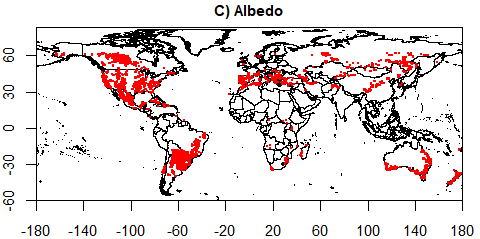


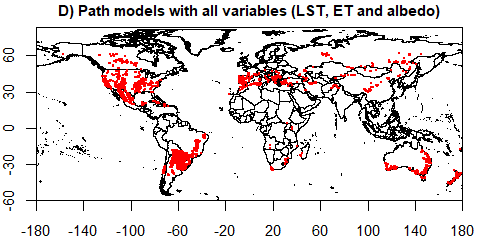


**S5 Fig. Spatial distribution of all valid pairs of focal/reference cells used in the analyses.** Each pair is represented as a single red point. A) Pairs used in the analyses of annual land surface temperature (LST; N = 36,493). B) Pairs used in the analyses of evapotranspiration (ET; N = 97,618). C) Pairs used in the analyses of albedo (N = 14,869). D) Pairs used in the path analyses, which contained valid information for the three climatic variables of interest (annual LST, ET and albedo ; N = 8,419).
