# Supplementary material for: Impacts of forestation and deforestation on local temperature across the globe
Source: PLoS One. 2019 Mar 20;14(3):e0213368. doi: 10.1371/journal.pone.0213368 (PMC6426338; doi:10.1371/journal.pone.0213368)
Supplement: S6 Fig — Four variables are shown: forest cover (first column), annual land surface temperature (LST, second column), evapotranspiration (ET, third column) and albedo (fourth column). Values for each variable were recorded for each pair of focal/reference cells, as the one-decade change observed in the focal cell minus the one-decade change in the reference cell (2010–2000 for forest cover, 2011–2001 for climatic variables). Values are shown separately for each region (Tropical, Temperate and Boreal) and for all regions combined (World). The blue vertical line indicates no difference between the focal and the reference cell in their one-decade change value (standardized change value = 0). (DOCX) [file pone.0213368.s006.docx]

|  |  | **Forest cover** | **Annual LST** | **ET** | **Albedo** |
| --- | --- | --- | --- | --- | --- |
| **Tropical** | Number of focal/reference pairs |  |  |  |  |
| **Temperate** |  |  |  |  |  |
| **Boreal** |  |  |  |  |  |
| **World** |  |  |  |  |  |
|  |  | Forest change (%) | LST change (ºC) | ET change (mm/month) | Albedo change (mm/month) |

**S6 Fig. Histograms of standardized change values used in the analyses.** Four variables are shown: forest cover (first column), annual land surface temperature (LST, second column), evapotranspiration (ET, third column) and albedo (fourth column). Values for each variable were recorded for each pair of focal/reference cells, as the one-decade change observed in the focal cell minus the one-decade change in the reference cell (2010 – 2000 for forest cover, 2011 – 2001 for climatic variables). Values are shown separately for each region (Tropical, Temperate and Boreal) and for all regions combined (World). The blue vertical line indicates no difference between the focal and the reference cell in their one-decade change value (standardized change value = 0).
