# Supplementary material for: Impacts of forestation and deforestation on local temperature across the globe
Source: PLoS One. 2019 Mar 20;14(3):e0213368. doi: 10.1371/journal.pone.0213368 (PMC6426338; doi:10.1371/journal.pone.0213368)
Supplement: S7 Fig — Effects of forest change on daytime (a) and nighttime (b) temperature. Each cell in the plots represents decadal (2011–2001) changes in annual means of climatic variables calculated for 0.05 x 0.05° cells grouped into bins of 5° latitude and 10% forest change. Positive (negative) values indicate a warming (cooling) effect of forest change. (DOCX) [file pone.0213368.s007.docx]

|  |  |
| --- | --- |

S7 Fig. Effects of forest change on daytime (a) and nighttime (b) temperature. Each cell in the plots represents decadal (2011 – 2001) changes in annual means of climatic variables calculated for 0.05 x 0.05º cells grouped into bins of 5º latitude and 10% forest change. Positive (negative) values indicate a warming (cooling) effect of forest change.
