# Supplementary material for: Impacts of forestation and deforestation on local temperature across the globe
Source: PLoS One. 2019 Mar 20;14(3):e0213368. doi: 10.1371/journal.pone.0213368 (PMC6426338; doi:10.1371/journal.pone.0213368)
Supplement: S8 Fig — Histograms of daytime (left) and nighttime (right) land surface temperature (LST) change values. Values were recorded for each pair of focal/reference cells, as the one-decade (2010–2000) change observed in the focal cell minus the one-decade change in the reference cell. Values are shown separately for each region (Tropical, Temperate and Boreal) and for all regions combined (World). The blue vertical line indicates no difference between the focal and the reference cell in their one-decade change value (standardized change value = 0). (DOCX) [file pone.0213368.s008.docx]

|  |  | **Daytime LST** | **Nighttime LST** |
| --- | --- | --- | --- |
| **Tropical** | Number of focal/reference pairs |  |  |
| **Temperate** |  |  |  |
| **Boreal** |  |  |  |
| **World** |  |  |  |
|  |  | LST change (ºC) | LST change (ºC) |

**S8 Fig. Histograms of daytime (left) and nighttime (right) land surface temperature (LST) change values.** Values were recorded for each pair of focal/reference cells, as the one-decade (2010 – 2000) change observed in the focal cell minus the one-decade change in the reference cell. Values are shown separately for each region (Tropical, Temperate and Boreal) and for all regions combined (World). The blue vertical line indicates no difference between the focal and the reference cell in their one-decade change value (standardized change value = 0).
