# Supplementary material for: Impacts of forestation and deforestation on local temperature across the globe
Source: PLoS One. 2019 Mar 20;14(3):e0213368. doi: 10.1371/journal.pone.0213368 (PMC6426338; doi:10.1371/journal.pone.0213368)
Supplement: S9 Fig — Histograms of standardized forest change values for deforestation (A) and forestation (B). These values were used in the analyses comparing effects of deforestation and forestation on annual land surface temperature change (see Fig 4 in the main text). (DOCX) [file pone.0213368.s009.docx]

| A) | B) |
| --- | --- |
|  |  |

**S9 Fig. Histograms of standardized forest change values for deforestation (A) and forestation (B).** These values were used in the analyses comparing effects of deforestation and forestation on annual land surface temperature change (see Fig. 4 in the main text).
