# Supplementary material for: Impacts of forestation and deforestation on local temperature across the globe
Source: PLoS One. 2019 Mar 20;14(3):e0213368. doi: 10.1371/journal.pone.0213368 (PMC6426338; doi:10.1371/journal.pone.0213368)
Supplement: S1 Table — Models were fit separately for each climatic variable (a—e), for each region (tropical, temperate or boreal*), and for each category of forest change (deforestation or forestation**). All models included the type of cell (reference or focal) as a fixed effect, and the searching window as a random effect. To account for spatial autocorrelation, different correlation structures were tested for each model, and the most suitable correlation structure was determined via model selection. Average, 95% confidence intervals and p-values were obtained from the top-ranked model only. N = number of focal/reference pairs of cells; t = t-statistic; p = significance level. Tests with p ≤ 0.05 are highlighted in bold. *Tropical = 20°S—20°N; Temperate = 20°S—50°S and 20°N—50°N; Boreal = >50°S and >50°N. **Deforestation and forestation correspond to decreases and increases in forest cover > 15%, respectively, from 2000 to 2010. ***Correlation structure of the top-ranked model. The correlation structures tested were corSpher, corLin, corRatio, corGaus,and corExp, with form = ~ longitude + latitude as implemented in the R package “nlme” (51). (DOCX) [file pone.0213368.s010.docx]

S1 Table. Summary of tests evaluating the effects of forest change on local climate.

| **Region** | **Deforestation** | | | | |  | **Forestation** | | | | |
| --- | --- | --- | --- | --- | --- | --- | --- | --- | --- | --- | --- |
|  | **Correlation structure***** | **Average (95% CI)** | ***N*** | ***t*** | ***P*** |  | **Correlation structure** | **Average (95% CI)** | ***N*** | ***t*** | ***P*** |
| *a) Change in annual temperature (ºC) (****Warmer****/* ***Cooler****)* | | | | | | | | | | | |
| Tropical | corRatio | **0.38 (0.35 – 0.41)** | 1725 | 25.67 | <0.001 |  | corGaus | **-0.18 (-0.23 – -0.14)** | 629 | -8.11 | <0.001 |
| Temperate | corExp | **0.16 (0.15 – 0.17)** | 16701 | 46.31 | <0.001 |  | corExp | **-0.19 (-0.20 – -0.17)** | 4538 | -29.50 | <0.001 |
| Boreal | corRatio | **-0.04 (-0.04 – -0.03)** | 9188 | -11.62 | <0.001 |  | corExp | **0.01 (-0.002 – 0.02)** | 3712 | 2.40 | 0.02 |
| World | corLin | **0.11 (0.10 – 0.11)** | 27614 | 40.75 | <0.001 |  | corRatio | **-0.10 (-0.11 – -0.09)** | 8879 | -23.70 | <0.001 |
|  | | | | | | | | | | | |
| *b) Change in evapotranspiration (mm/month) (****Dryer****/* ***Wetter****)* | | | | | | | | | | | |
| Tropical | corGaus | **-6.18 (-6.34 – -6.03)** | 20348 | -76.40 | <0.001 |  | corGaus | **7.04 (6.62 – 7.47)** | 4603 | 32.81 | <0.001 |
| Temperate | corGaus | **-2.50 (-2.56 – -2.44)** | 25890 | -82.56 | <0.001 |  | corRatio | **5.33 (5.14 – 5.53)** | 5791 | 53.47 | <0.001 |
| Boreal | corGaus | **-2.34 (-2.37 – -2.30)** | 27666 | -138.41 | <0.001 |  | corExp | **2.07 (2.02 – 2.12)** | 13320 | 82.40 | <0.001 |
| World | corLin | **-3.46 (-3.51 – -3.41)** | 73904 | -131.60 | <0.001 |  | corLin | **3.82 (3.72 – 3.92)** | 23714 | 73.14 | <0.001 |
|  | | | | | | | | | | | |
| *c) Change in albedo (%) (****Brighter****/* ***Darker****)* | | | | | | | | | | | |
| Tropical | corRatio | **0.82 (0.75 – 0.90)** | 692 | 22.16 | <0.001 |  | corExp | **0.16 (0.01 – 0.31)** | 166 | 2.11 | 0.04 |
| Temperate | corRatio | **0.97 (0.93 – 1.00)** | 8485 | 53.04 | <0.001 |  | corRatio | **-0.70 (-0.74 – -0.65)** | 2937 | -32.19 | <0.001 |
| Boreal | corExp | **4.25 (4.13 – 4.38)** | 2197 | 68.26 | <0.001 |  | corRatio | **-1.95 (-2.19 – -1.72)** | 392 | -16.34 | <0.001 |
| World | corRatio | **1.60 (1.55 – 1.64)** | 11374 | 72.39 | <0.001 |  | corRatio | **-0.80 (-0.85 – -0.75)** | 3495 | -32.82 | <0.001 |
|  | | | | | | | | | | | |
| *d) Change in daytime temperature (ºC) (****Warmer****/* ***Cooler****)* | | | | | | | | | | | |
| Tropical | corExp | **0.86 (0.84 – 0.88)** | 10464 | 84.95 | <0.001 |  | corLin | **-0.48 (-0.53 – -0.44)** | 2192 | -22.15 | <0.001 |
| Temperate | corGaus | **0.45 (0.44 – 0.46)** | 21104 | 77.38 | <0.001 |  | corExp | **-0.49 (-0.52 – -0.47)** | 5380 | -40.00 | <0.001 |
| Boreal | corExp | **0.16 (0.15 – 0.17)** | 13412 | 40.35 | <0.001 |  | corRatio | **-0.07 (-0.08 – -0.05)** | 5631 | -11.32 | <0.001 |
| World | corGaus | **0.46 (0.45 – 0.46)** | 44980 | 115.24 | <0.001 |  | corExp | **-0.31 (-0.32 – -0.29)** | 13203 | -44.63 | <0.001 |
|  | | | | | | | | | | | |
| *e) Change in nighttime temperature (ºC) (****Warmer****/* ***Cooler****)* | | | | | | | | | | | |
| Tropical | corRatio | 0.00 (-0.02 – 0.01) | 3185 | -0.44 | 0.66 |  | corRatio | **0.10 (0.07 – 0.13)** | 1735 | 6.94 | <0.001 |
| Temperate | corGaus | **-0.15 (-0.15 – -0.14)** | 21796 | -44.67 | <0.001 |  | corLin | **0.11 (0.10 – 0.13)** | 5595 | 16.16 | <0.001 |
| Boreal | corGaus | **-0.25 (-0.25 – -0.24)** | 23641 | -80.07 | <0.001 |  | corExp | **0.07 (0.06 – 0.08)** | 10843 | 16.84 | <0.001 |
| World | corLin | **-0.19 (-0.19 – -0.18)** | 48622 | -85.10 | <0.001 |  | corExp | **0.07 (0.06 – 0.08)** | 18173 | 24.27 | <0.001 |

Models were fit separately for each climatic variable (a - e), for each region (tropical, temperate or boreal*), and for each category of forest change (deforestation or forestation**). All models included the type of cell (reference or focal) as a fixed effect, and the searching window as a random effect. To account for spatial autocorrelation, different correlation structures were tested for each model, and the most suitable correlation structure was determined via model selection. Average, 95% confidence intervals and P-values were obtained from the top-ranked model only. N = number of focal/reference pairs of cells; t = t-statistic; P = significance level. Tests with P ≤ 0.05 are highlighted in bold.

*Tropical = 20ºS-20ºN; Temperate = 20ºS-50ºS and 20ºN-50ºN; Boreal = >50ºS and >50ºN

**Deforestation and forestation correspond to decreases and increases in forest cover > 15%, respectively, from 2000 to 2010.

***Correlation structure of the top-ranked model. The correlation structures tested were corSpher, corLin, corRatio, corGaus,and corExp, with form = ~ longitude +latitude as implemented in the R package “nlme” (*51*).
