# Supplementary material for: Impacts of forestation and deforestation on local temperature across the globe
Source: PLoS One. 2019 Mar 20;14(3):e0213368. doi: 10.1371/journal.pone.0213368 (PMC6426338; doi:10.1371/journal.pone.0213368)
Supplement: S2 Table — Coefficients are presented for each link of the path model (“Response”—“Predictor” pair). Models were fit separately for tropical, temperate and boreal regions. Three alternative spatial correlation structures were tested: rational quadratic (corRatio), exponential (corExp) and gaussian (corGaus). LST = change in annual land surface temperature; ET = change in evapotranspiration; Forest = change in forest; Albedo = change in albedo. NS = statistically non-significant coefficients (p > 0.05). See also Fig 4 in the main text for a graphical presentation of the coefficients of the model with corRatio structure. (DOCX) [file pone.0213368.s011.docx]

**S2 Table.** Standardized coefficient estimates for multi-level structural equation models.

| **Response** | **Predictor** | **corRatio** | **corExp** | **corGaus** |
| --- | --- | --- | --- | --- |
| *a) Tropical region* |  |  |  |  |
| LST | ET | -0.51 | -0.52 | -0.51 |
| LST | Albedo | 0.14 | 0.13 | 0.15 |
| LST | Forest | -0.09 (NS) | -0.09 (NS) | -0.09 (NS) |
| Albedo | Forest | -0.43 | -0.44 | -0.42 |
| ET | Forest | 0.64 | 0.63 | 0.62 |
| ET | Albedo | 0.16 | 0.14 | 0.17 |
|  |  |  |  |  |
| *b) Temperate region* |  |  |  |  |
| LST | ET | -0.37 | -0.37 | -0.36 |
| LST | Albedo | 0.09 | 0.09 | 0.11 |
| LST | Forest | -0.21 | -0.22 | -0.20 |
| Albedo | Forest | -0.49 | -0.49 | -0.48 |
| ET | Forest | 0.61 | 0.62 | 0.61 |
| ET | Albedo | 0.06 | 0.06 | 0.06 |
|  |  |  |  |  |
| *c) Boreal region* |  |  |  |  |
| LST | ET | -0.06 (NS) | -0.07 (NS) | -0.06 (NS) |
| LST | Albedo | -0.31 | -0.29 | -0.32 |
| LST | Forest | -0.21 (NS) | -0.19 (NS) | -0.21 (NS) |
| Albedo | Forest | -0.73 | -0.72 | -0.73 |
| ET | Forest | 0.95 | 0.93 | 0.97 |
| ET | Albedo | 0.16 | 0.13 | 0.17 |

Coefficients are presented for each link of the path model (“Response” - “Predictor” pair). Models were fit separately for tropical, temperate and boreal regions. Three alternative spatial correlation structures were tested: rational quadratic (corRatio), exponential (corExp) and gaussian (corGaus). LST =change in annual land-surface temperature; ET = change in evapotranspiration; Forest = change in forest; Albedo = change in albedo. NS = statistically non-significant coefficients (*P*> 0.05). See also Fig. 3 in the main text for a graphical presentation of the coefficients of the model with corRatio structure.
